# Experimental study on healing of long bone defects treated with fibrin membrane enriched with platelet growth factors and periosteal mesenchymal stem cells in rabbit: Radiographical and histopathological evaluations

**DOI:** 10.30466/vrf.2018.86692.2124

**Published:** 2019-12-15

**Authors:** Amin Paidar Ardakani, Mohammad Mehdi Oloumi, Alireza Farsinejad, Reza Kheirandish

**Affiliations:** 1 *Department of Clinical Sciences, Faculty of Veterinary Medicine, Ardakan University, Ardakan, Iran;*; 2 *Department of Clinical Sciences, Faculty of Veterinary Medicine, Shahid Bahonar University of Kerman, Kerman, Iran;*; 3 *Department of Hematology and Medical Laboratory Sciences, Faculty of Allied Medicine, Kerman University of Medical Science, Kerman, Iran;*; 4 *Department of Pathobiology, Faculty of Veterinary Medicine, Shahid Bahonar University of Kerman, Kerman, Iran.*

**Keywords:** Bone healing, Fibrin membrane, Platelet growth factor, Rabbit, Stem cell

## Abstract

The present study was designed to evaluate the effects of platelet growth factors and periosteal mesenchymal stem cells on bone healing process, radiographically. Forty male White New Zealand rabbits in five equal groups were used in this study. A 2 mm full thickness bone defect was made in left radial bone of each animal. In group A (control) the defect was left with no medical intervention. In group B the defect was covered by a fibrin membrane. In group C the defect was covered by a fibrin membrane plus platelet growth factors. In group D the defect was covered by a fibrin membrane plus periosteal mesenchymal stem cells, and in group E the defect was covered by a fibrin membrane enriched with platelet growth factors and periosteal mesenchymal stem cells. Radiological evaluation was done immediately after surgery (week 0) and then at the 1^st^, 2^nd^, 4^th^, 6^th^ and 8^th^ weeks after operation. At the end of the eighth week, bone samples were taken to evaluate the histopathology. The radiological and histopathological observations showed a superior bone healing in the groups D and E, after eight weeks in comparison with the groups A, B and C. According to this study, it could be concluded that the platelet growth factors and periosteal mesenchymal stem cells could promote bone regeneration in long bone defects in a rabbit model.

## Introduction

Bone is a very strong and rigid protective organ of the animals and human being. They are found in different shapes and forms having complex internal and external structures. They are of prime importance as they provide the major locomotor structural and supportive elements to the animals. Bone fracture occurs commonly due to different accidents and injuries.^1^ Fracture healing is a complex, well-organized regenerative process initiated in response to injury, which results in optimal skeletal repair and restoration of skeletal function. Of the estimated 7.90 million fractures that occur annually in the United States, approximately 5.00 to 10.00% have either delayed or impaired healing.^2^ Also, of these 600,000 fractures with delayed healing, nearly 100,000 end up nonunion.^3^ The healing process involves the coordinated participation of hematopoietic and immune cells within the bone marrow, and vascular and skeletal cell precursors including mesenchymal stem cells.^4^ Bone tissue engineering, which combines the application of the principles of orthopedic surgery with basic science and engineering, has been heralded as an alternative when bone regeneration is required to replace or restore the function of traumatized, damaged or lost bone.^5^ Platelets, also called thrombocytes, are a component of blood whose function, along with the coagulation factors, is to stop bleeding by clumping and clotting blood vessel injuries.^6^ Platelets do not have cell nucleus, they are fragments of cytoplasm that are derived from the megakaryocytes of the bone marrow, and then enter the circulation.^7^ The main function of platelets is to contribute to hemostasis, the process of stopping bleeding at the site of interrupted endothelium.^8^ Platelets regenerative potential was reported in the 70’s,^9^ when it was observed that they contain growth factors responsible for increase collagen production, cell mitosis, blood vessels growth, recruitment of other cells that migrate to the site of injury and cell differentiation induction, among others.^10^ Platelets have an important role in the complex local inflammatory response at sites of bone healing^11^ where, on activation, platelets promote angiogenesis,^12^ recruit mesenchymal cells^13^ and are a source of growth factors (GFs) required in bone healing. Nowadays in oral surgery there are two kinds of platelet concentrates for *in vivo* tissue engineering applications: platelet-rich plasma (PRP) and platelet-rich fibrin (PRF). Platelet concentrates are a concentrated suspension of growth factors found in platelets, which act as bioactive surgical additives that are applied locally to induce wound healing.^14^ The PRF consists of a matrix of autologous fibrin^15^ and has several advantages over PRP, including easier preparation and not requiring chemical manipulation of the blood, which makes it strictly an autologous preparation.^16^ For these considerations, in our study we preferred to use PRF procedure instead of PRP. The PRF is an autologous fibrin matrix containing platelets and leukocyte growth factors, which are obtained from inside the fibrin clot, which may explain the slow release of bioactive proteins (growth factors) from the PRF.^17^^-^^19^ The platelet count in PRF is three-to-seven times greater than its normal concentration in blood. Growth factors obtained from PRF include platelet-derived growth factor (PDGF), transforming growth factor-β (TGF- β), and insulin-like growth factor (IGF).^18^^,^^20^ The PRF consists of an autologous leukocyte-platelet-rich fibrin matrix^21^ composed of a tetra molecular structure, with cytokines, platelets and stem cells within it^15^^,^^22^ which acts as a biodegradable scaffold^23^ that favors the development of microvascularization and is able to guide epithelial cell migration to its surface.^15^^,^^24^ Some studies have demonstrated that PRF is a healing biomaterial with a great potential for bone and soft tissue regeneration without inflammatory reactions and may be used alone or in combination with bone grafts, promoting hemostasis and bone growth and maturation.^25^^,^^26^ The PRF was first used specifically in oral surgery by Dohan *et al*. and is currently considered as a new generation of platelet concentrate.^17^ This autologous matrix in the *in vitro* studies has been demonstrated to bear a great potential to increase cell attachment and a stimulation to proliferate and differentiate osteoblasts. In surgical procedures, PRF could serve as a resorbable membrane for guided bone regeneration (GBR), preventing the migration of non-desirable cells into bone defect and providing a space that allows the immigration of osteogenic and angiogenic cells permitting the underlying blood clot to mineralize. Moreover, a normal PRF membrane has a rapid degradability (1 to 2 weeks).^27^ Stem cells are promising tools for studying the mechanisms of development and regeneration and for use in cell therapy of various disorders. They have been identified in most organ tissues and therefore specifically named as hematopoietic, neural, gastrointestinal, epidermal, hepatic and mesenchymal stem cells (MSCs). The MSCs show a high capacity of self-renewal and differentiation in to various lineages and they have been mostly used for the biological repair of cartilage and bone. The MSCs, firstly identified by Ferretti *et al*. in bone marrow (BM), have been also isolated from many other tissues, including peripheral blood, amniotic fluid, adipose tissue, skin, skeletal muscle, fetal tissues, synovial membrane, articular cartilage and compact bone. However, cells from diverse sources may show phenotypic heterogeneity and particularly different *in vivo* results and/or specific functions of their graft regions after transplantation.^28^ Hence, a correct selection of MSCs source is crucial to obtain a more efficient treatment for regeneration of injured osteochondral tissues. In particular the periosteum, a thin tissue that covers the outer cortical bone surface, contains a reservoir of progenitor cells that contribute to bone repair. Periosteum-derived cell preparations can form cartilage and bone both *in vitro* and *in vivo*.^29^

The aim of this experimental study was to evaluate the quality and speed of bone repair of rabbits by simultaneous use of PRF prepared from human blood (hPRF) and enriched with GFs in combination with MSCs isolated from the periosteal bone of the rabbit.

## Materials and Methods

**Animals. **In this study, forty healthy male adult New Zealand White rabbits with a weight range of 2.50 to 3.00 kg were randomly assigned into five equal groups. Rabbits were individually housed in metallic cages in air-conditioned room (12 hr light-dark cycles with a temperature of 21.00 ˚C and relative humidity of 50.00%) in the laboratory animal center of Shahid Bahonar University of Kerman. The animals had free access to water and platted food. All the procedures were conducted in accordance with the European Community guidelines for laboratory animals and under the supervision of the Ethic Committee of the Faculty of Veterinary Medicine, Shahid Bahonar University of Kerman (2010/63/EU).

**Surgical procedure.** To create bone defect, rabbits were anesthetized with an intramuscular injection of 50.00 mg kg^-1^ ketamine hydrochloride (Alfasan, Woerden, Netherlands) and 5.00 mg kg^-1^ xylazine (Alfasan). The anesthesia was maintained under isoflurane (Piramal Healthcare, Gujarat, India) and oxygen, applied through a face mask from the anesthetic machine (DM6a; Shanghai, China). The left antebrachium of each animal was prepared for surgery in standard manner. The left radius was approached craniomedially and a two mm full-thickness bone defect was made at the mid shaft by an oscillating saw (Leytemed, Guangdong, China). Following the application of the treatment materials according to the grouping of the animals the wounds were closed routinely. 

**Preparation of fibrin membrane. **Fibrin membrane was prepared using human cryopercipitate product. For this purpose, Cryo (Cryo Products, Hambakenwetering, Netherlands) was first melted and was admixed with equal volumes of saline and cold ethanol (Zibo Aojin Chemical Co., Zibo, China). After the formation of insoluble white color of fibrinogen, the pure fibrinogen was precipitated via centrifuging and the liquid was discarded. In the next step, by adding sodium citrate 3.20% (Willpowder, Miami, USA) the sediment was dissolved and then by adding human thrombin in a sterile plain glass tube, fibrinogen was polymerized and turned into fibrin. The sediment was eventually clogged and membrane-shaped.

**Preparation of platelet growth factors. **Growth stimulants were obtained from human platelets. For this purpose, a platelet concentrate bag from the Blood Transfusion Organization of Iran (Tehran, Iran) was received. The content of the bag was poured into a 50 mL flask under sterile condition and centrifuged at 3500 rpm for 15 min (Eppendorf, New Delhi, India). Platelet sediments, along with a small amount of plasma, were kept in the 50 mL flask and the supernatant solution was discarded. By adding 1.00 mL of human thrombin and 10.00% calcium chloride (Will powder), the platelets were activated. In the next step, the platelets extract was isolated by centrifugation and used as a source of platelet growth factors. For enrichment, the human fibrin membrane was impregnated in growth factors.

**Preparation of periosteal mesenchymal stem cells. **In order to obtain allogeneic periosteum, a New Zealand rabbit as donor was anesthetized and prepared for operation. The radius was approached and a 0.50 × 1.00 cm piece of periosteum was harvested from the bone with a periosteal elevator under sterile condition, and cultured in a basic culture medium (DMEM-F12; Merck, Darmstadt, Germany).

In order to separate the stem cells, the periosteum was washed with phosphate buffer saline (PBS; Boston Bio Products, Ashland, USA) and cut into several pieces with a sterile surgical blade. The pieces were placed in a 2.00% type 1 collagenase solution (Stemcell Technologies, Vancouver, Canada) for 30 min. The solution was then passed through a 122 µm millipore filter (Merck). The equal volume of PBS was then added to the solution and the resulting mixture was centrifuged for at 1200 rpm for 1 min. Then, 5000 cells per square centimeter were cultured from a 75 cm square flask. The culture medium of these cells was DMEM-F12 containing 12.00% bovine serum (Biowest, Bourges, France), penicillin (Jaber Ebne Hayyan, Tehran, Iran) and streptomycin (Jaber Ebne Hayyan). Plate containing cells was transferred to a 37.00 ˚C incubator containing 5.00% CO_2_, and after the appearance of spiky colonies, the medium was replaced.

**Animal grouping.** Group A (control): The bone defect was left with no treatment. Group B: Fibrin membrane, in which fibrin membrane strip was wrapped around the bone defect. Group C: Fibrin membrane plus platelet growth factors. The fibrin membrane strip enriched with platelet growth factors was used at the site of bone defect. Group D: Fibrin membrane plus periosteal mesenchymal stem cells. The fibrin membrane strip containing periosteal mesenchymal stem cells was used at the site of bone defect. Group E: Fibrin membrane plus platelet growth factors and periosteal mesenchymal stem cells. The fibrin membrane strip enriched with GFs and MSCs was used at the site of bone defect. Following surgery, the limb was fixed in fiberglass cast (Bandhaye Pezeshki Iran Co., Tehran, Iran) for two weeks. Pantrisul^®^ (trimethoprim and sulfa-methoxazole; 30.00 mg kg^-1^, bid, IM; Pantex Co., Hapert, Netherlands) was administered for five days to prevent the infection. Tramadol (5.00 mg kg^-1^, bid, IM; Aburaihan Co., Tehran, Iran) was also administered for two days to control pain. 

**Histopathological evaluation. **Immediately after preparation, samples were fixated in formalin buffer solution 10.00% (Pars Tousheh, Tehran, Iran). The formalin solution was replaced after 24 hr. The samples were cleansed from soft tissue and muscle, and decalcified with nitric acid 15.00% (Karun Petrochemical Co., Tehran, Iran). They were then placed in Autotechnicon (Yu Shuo Da, Shenyang, China). The specimens were molded with paraffin and sections of thickness of 5.00 µm were prepared. Hematoxylin and Eosin were used for staining. Finally, in order to examine the healing process and the quality of the callus, the slides were studied under light microscope (Motic-Ted Pella, Redding, USA) qualitatively.^30^

**Radiographical evaluation. **For radiographical evaluations, two perpendicular views of the operated limb (dorsopalmar, and lateral views) were taken immediately and on weeks 1, 2, 4, 6, and 8 after surgery. Parameters to be evaluated in the radiographs included. Callus formation, size of the gap at the osteotomy site, and quality of the bone repair tissue. For quantitative evaluation of the radiographs, the RUST scoring system (radiographic union scour for tibia) was used (Table 1). This scoring system is based on the quantity of bone callus formation and the observation of the fracture line in the two lateral and dorsopalmar radiographic views in the four bone cortex which is specified in the radiographs. Each cortex was assigned a RUST score of 1 to 3, based on the appearance In which a cortex with a visible fracture line and no callus was given a score of 1, a cortex where callus and a visible fracture line was present was scored as 2, and a cortex with bridging callus and no fracture line within the callus bridge was scored as 3.^31 ^Finally, the scores for each of the four cortices were grouped according to the Table 1 and the RUST index was determined for each case.

**Table 1:** The radiographic union scale in tibial fracture (RUST).

**Score per cortex**	**Callus**	**Fracture Line**
**1**	Absent	Visible
**2**	Present	Visible
**3**	Present	Invisible

**Statistical analysis. **The data were analyzed by the non-parametric Mann-Whitney U statistical tests among the groups, using SPSS (version 16; IBM, Chicago, USA), and a *p ≤* 0.05, was considered as significant.

## Results

There was no intraoperative and postoperative death during the study. None of the rabbits showed wound infection, surgery complication or ulnar bone fracture at the radial bone defect. 

**Radiographic evaluation. **The results of radiological evaluations at on weeks 1, 2, 4, 6, and 8 after surgery are presented in Table 2.

**Fig. 1:**
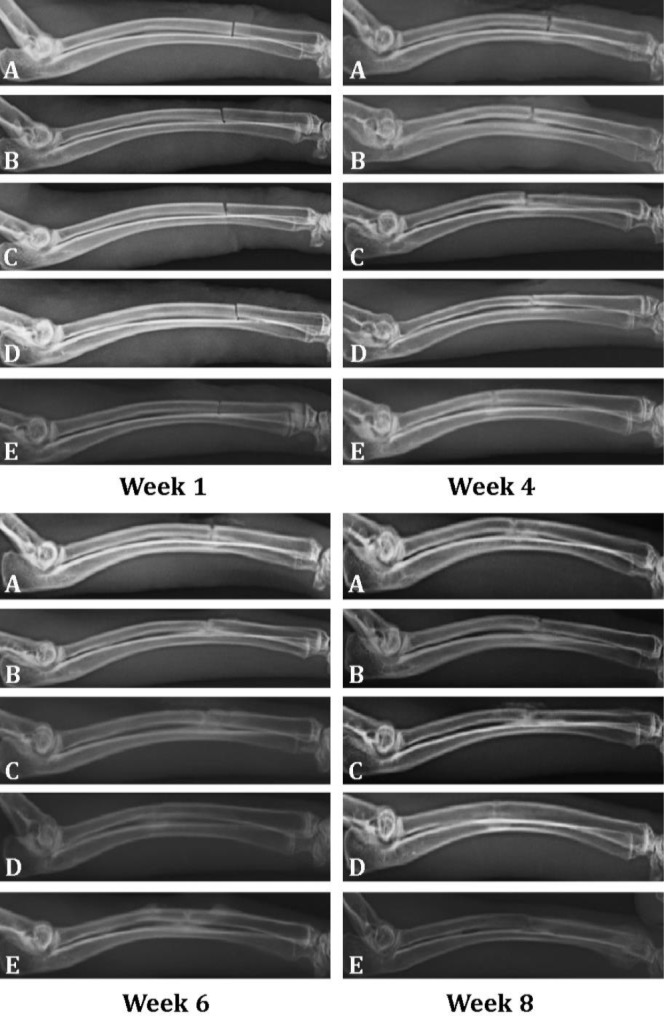
Radiographs of radius at 1, 4, 6, and 8 weeks post-operation. **A)** Group A (control): The bone defect was left with no treatment; **B)** Group B: fibrin membrane; **C)** Group C: fibrin membrane plus platelet growth factors, **D)** Group D: fibrin membrane plus periosteal mesenchymal stem cells; and **E)** Group E: fibrin membrane plus platelet growth factors and periosteal mesenchymal stem cells

The analysis of data showed that there were no significant differences in healing of the bone defect, based on radiological scores among the animals of all five groups on week 1, 2 and at 4 weeks post-injury (*p* > 0.05), ( Table 2, Fig. 1). However, at week 6 and 8 post-operation the radiographs showed significant differences among the groups (*p* ≤ 0.05), and the differences at 6 week were more prominent than those of the week 8 (Table 2). At week 6, the bone union and callus formation in the group D was significantly better than the groups B, C and E (*p* ≤ 0.05). Also, there was a significant difference in bone healing process between groups B and C at week 6^th^, so that, the performance of group C was better (*p* ≤ 0.05). 

At week 8, the performance of group D was better than groups C and B, however, the difference was close to meaningful (*p* = 0.061 and *p* = 0.064, respectively). Our data analysis showed that the best effect was seen in group D. 

**Table 2:** Radiographical findings for healing of the bone defect (n = 8) at various post-operative intervals. Data are presented as median (min-max)

**Weeks post-operation**	**Group A**	**Group B**	**Group C**	**Group D**	**Group E**
**0**	4(4-4)	4(4-4)	4(4-4)	4(4-4)	4(4-4)
**1**	4(4-5)	5(4-6)	4(4-6)	4(4-5)	5(4-6)
**2**	7.50(7-8)	8(8-8)	8(6-8)	7.50(7-8)	7(6-8)
**4**	8(7-8)	8(8-8)	8(8-8)	8.50(8-9)	8(8-10)
**6**	10(9-12)	8(8-9)	10(10-10)^*^	11(11-11)^† α β^	10(8-11)
**8**	10(9-12)	9(8-11)	10.50(10-11)	11.50(11-12)	11(10-12)

^* ^indicates significant difference compared to group B at *p* = 0.018, ^†^ indicates significant difference compared to group B* p* = 0.018, ^α^ indicates significant difference compared to group C at* p* = 0.008, and ^β^ indicates significant difference compared to group E at* p* = 0.031.

**Fig. 2:**
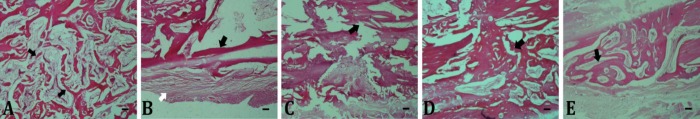
**A)** The presence of bone callus with the arrangement of thin and irregular trabeculae (black arrow); **B)** Note bone callus (black arrow) accompanied by an inflammatory reaction (white arrow) at the site of the lesion; **C)** The presence of bone callus with the arrange-ment of thin and irregular trabeculae (black arrow); **D)** Adult and regular bone callus formation without any inflammatory reaction in group D (black arrow); and **E)** Note adult bone callus without any inflammatory reaction in group E (black arrow), (H & E, Bar = 100 µm)

**Histopathological evaluations. **Control group: Bone callus formed in the defect space had thin trabeculae that were discontinuous in some areas (Fig. 2A). Group B: In the presence of bone callus in the bone cortex and periosteum region, an inflammatory reaction was observed (Fig. 2B). Group C: The bony trabeculae formed in the bone defect area were thin and discontinuous similar to the control group (Fig. 2C). Groups D and E: Bone callus had thick and mature trabeculae. The boundary between the reconstructive bone and the original bone was clear. Nevertheless, there was a more organized callus in group D. None of these two groups had an inflammatory reaction (Figs. 2D and 2E).

 According to the results of histopathology, adult bone callus formation and absence of inflammatory reactions, the best performance was observed in group D and in the next in group E.

## Discussion

The objective of this study was to evaluate the bone repair effects of hPRF enriched with platelet growth factors in combination with mesenchymal stem cells isolated from the periosteal bone of the rabbit. The radial bone defect of rabbits is a convenient model for study of bone-regenerative materials because of its lack of fixation requirements.^32^ Small rodents have primitive bone structures and do not have Haversian systems and though little is known about the importance of this anatomical difference between rodents and humans and this makes bone repair in these animals different from that seen in human beings. Whereas, rabbits as well as caprines and dogs, have Haversian systems that are similar to that of human which is an important advantage in terms of extrapolation of results obtained from such animals for human bone repair.^33^

Several laboratory and clinical studies have been conducted on the effects of fibrin membrane, platelet growth factors and stem cells on the process of long bone repair. Most of these studies point to their positive effects, although some of these studies suggest that they are not effective. It has been reported that injection of bone marrow cells at the site of infections nonunion of tibia could stimulate bone formation in these cases.^34^^,^^35^ Bajada *et al*. in a study on nine-year nonunion fracture that had a history of six surgeries, reported that the use of mesenchymal stem cells along with calcium sulfate powder caused bone union after two months post-surgery.^36^ To treat children with the progressive deformity osteogenesis imperfecta (OI), Horwitz *et al*. used a bone morrow transplant. After three months a dense bone was formed and total-body bone mineral content was increased, and reduced frequency of bone fractures with an increased growth velocity was observed. That study implied that “mesenchymal progenitors” in transplanted marrow resulted in improved bone quality in patients with OI.^37^

Some advantages have been reported in the literature related to the use of PRF: Its preparation is simple and efficient with centrifugation in a single step and accessibility for all clinicians.^27^ It is obtained by autologous blood sample;^24^ and minimized blood manipulation is required.^27^ It does not require the addition of external thrombin because polymerization is a completely natural process, without any risk of suffering from an immunological reaction.^17^^,^^27^ It has a natural fibrin framework with growth factors within that may keep their activity for a relatively longer period and stimulate tissue regeneration effectively.^27^ It can be used solely or in combination with bone grafts, depending on the purpose. It increases the healing rate of the grafted bone.^22^^,^^27^ It is an affordable and quick option compared to recombinant growth factors when used in conjunction with bone grafts.^27^ The PRF membrane helps improve wound healing, protecting the surgical site, promoting soft tissue repair, when mixed with bone graft and it may act as a “biological connector” which attracts stem cells, favors the migration of oste-oprogenitor cells to the center of the graft and provides a neo-angiogenesis.^38^ In addition, PRF may act as a biologic adhesive to hold the particles together and facilitating the manipulation of the bone grafts.^39^ *In vitro* study showed that PRF has a strong potential for increasing cellular attachment and is a powerful stimulant for the proliferation and differentiation of osteoblasts.^18^^,^^27^ Schwartz-Arad *et al*. showed that the use of PRF with bone graft in the mandibular alveolar bone lesions can accelerate the repair process.^40^ In another study, Lee *et al*. compared autogenous grafts to autogenous graft plus PRF combinations for sinus-lifting operations. Based on a histomorphometric analysis, they found a greater amount of bone in the autogenous graft plus PRF combination group compared to the group treated with autogenous bone grafts alone.^41^

The results of the present study indicated that the combination of fibrin membrane and MSCs (group D) had more prominent effects on bone healing in comparison with the other groups. 

Platelet-rich fibrin seemed to assure a better soft tissue healing and making the wound healing faster.^22^ Moreover, several clinical works have demonstrated the effectiveness of PRF in promoting the healing of surgical wounds. The PRF has, in fact, platelet growth factors that can improve the vascularization of the surgical site and promote neoangiogenesis.^17^ One of these growth factors is transforming growth factor-β (TGF- β) which is a potent inhibitor of hemopoietic stem cell proliferation *in vivo* reported by others.^42^ It should be considered that differences in isolating PRF, the use of a specific anticoagulant that could affect platelet fragmentation and effectiveness of GF could be the most important variables affecting literature results.^43^ Weaker bone repair effects in groups C and E in comparison with group D could be attributed to the presence of growth factors in these two groups, though, these two groups showed a better bone healing than groups A and B. 

Finally, this study showed that the best effect was seen in group D and then in E. Based on radiological and histopathological evaluations in the present study, it can be concluded that periosteal mesenchymal stem cells alone or in combination with platelet growth factors can play a positive role in promoting bone healing characteristics compared to negative control and fibrin membrane control groups.
